# Hypoparathyroidism: State of the Art on Cell and Tissue Therapies

**DOI:** 10.3390/ijms221910272

**Published:** 2021-09-24

**Authors:** Francesca Miglietta, Gaia Palmini, Francesca Giusti, Simone Donati, Cinzia Aurilia, Teresa Iantomasi, Maria Luisa Brandi

**Affiliations:** 1 Department of Experimental and Clinical Biomedical Sciences, University of Florence, Viale Pieraccini 6, 50139 Florence, Italy; francesca.miglietta@unifi.it (F.M.); gaia.palmini@unifi.it (G.P.); francesca.giusti@unifi.it (F.G.); simone.donati@unifi.it (S.D.); cinzia.aurilia@unifi.it (C.A.); teresa.iantomasi@unifi.it (T.I.); 2 Fondazione Italiana Ricerca sulle Malattie dell’Osso (F.I.R.M.O Onlus), 50139 Florence, Italy

**Keywords:** stem cells, transplantation, hypoparathyroidism, cell therapy, tissue therapy

## Abstract

Hypoparathyroidism is an endocrine disorder characterized by low serum calcium levels, high serum phosphorus levels, and by inappropriate or absent secretion of the parathyroid hormone (PTH). The most common therapeutic strategy to treat this condition is hormone replacement therapy with calcium and vitamin D but, unfortunately, in the long term this treatment may not be sufficient to compensate for the loss of endocrine function. Glandular autotransplantation is considered the most effective technique in place of replacement therapy. Although it leads to excellent results in most cases, autotransplantation is not always possible. Allograft is a good way to treat patients who have not been able to undergo autograft, but this technique has limited success due to side effects related to tissue rejection. This therapy is supported by systemic immunosuppression, which leads to the onset of serious side effects in patients, with a risk of endocrine toxicity. Today, research on endocrine disorders is focused on discovering alternative graft therapies that can allow optimal results with the fewest possible side effects. In this review, we will make an update on the current state of the art about the cell and tissue therapy as treatment for hypoparathyroidism, to identify which type of therapeutic strategy could be valid for a future clinical use.

## 1. Introduction

The endocrine system consists of a group of glands and organs that regulate and control the various functions of the body through the production and secretion of hormones. Hormones act as messengers by controlling and coordinating activities throughout the body. The different types of dysfunctions can lead to a hormone overproduction (hyperfunction) or hormone deficiency (hypofunction). Due to the complexity of hormonal metabolic interactions, endocrine disorders are complicated diseases to treat [1,2,3,4,5].

In physiological conditions parathyroid glands have as their function the maintenance of iodized calcium levels in the blood, within a very narrow range. This regulation occurs trough minute-by-minute modulation of parathyroid hormone (PTH) release into the circulation. This modulation has an immediate effect on urinary calcium excretion and in bone calcium efflux. If this PTH modulation persists, there are changes in the metabolism of vitamin D at the renal level. Resulting from the alteration of vitamin D metabolism, we then have the efficiency of intestinal calcium via the absorption modulation [6,7,8].

Among endocrine disorders we find hypoparathyroidism (HypoPT). HypoPT is a rare condition characterized by low levels of calcium, high levels of phosphorus and an underproduction of PTH [6].

The clinical manifestations of hypocalcemia are the result of the degree of severity of the hypocalcemia itself, the age at onset, and the duration of the disease. Generally, people with chronic HypoPT complain about paresthesia, an unpleasant tingling sensation around the mouth, hands, and feet, as well as muscle cramps and severe spasms, up to tetanic contractions. Hypocalcemia has a profound impact on numerous tissues and organs, including the brain, muscles, heart, and kidneys. In fact, hypocalcemia can be the cause of medical emergencies, such as severe irregularities in normal heartbeat, seizures, laryngospasm, stridor, bronchospasm, wheezing, or it can develop gradually and be almost asymptomatic.

HypoPT can be caused by the defect of numerous genes and can arise as an isolated form, or associated with other endocrine and non-endocrine alterations (primary HypoPT) [6,7,8,9,10,11,12].

Genetic disorders can be divided in one group where HypoPT is caused by genetic alterations during the formulation of parathyroid glands, a second group characterized by the alteration of parathyroid gland secretion and a third group in which we find the congenital disorders of parathyroid glands. The first group recognizes disorders of the formation of the parathyroid glands [6,7,9,10,11,12]. This is a group of disorders in which the parathyroid glands do not form properly during embryonal development and consequently result in insufficient production of PTH. The phenotypic spectrum of HypoPT ranges from the absence of symptoms in carriers of the mutated gene to severe hypocalcemia and seizures. Although hypocalcemia is often present in childhood, symptoms may not be evident until adulthood (DiGeorge syndrome type 1 and 2; CHARGE syndrome, HypoPT, sensorineural deafness and renal dysplasia (HDR), Kenny-Caffey syndrome type 1 and 2, Mitochondrial Disorders Familial isolated hypoparathyroidism type 2, Hypoparathyroidism X-linked recessive]. The second group of genetic disorders includes disorders of parathyroid gland secretion [6,7,9,10,12,13]. These include diseases with reduced sensitivity of the calcium-sensitive apparatus and mutations in the PTH gene itself [Autosomal dominant hypocalcemia with hypercalciuria type 1 (ADH1) and type 2 (ADH2); Familial isolated hypoparathyroidism type 1 (FIH) and Hypomagnesemia syndromes]. The third group of congenital disorders includes disorders of damage to the parathyroid glands [6,7,9,10,11,12]: autoimmune hypoparathyroidism, which can manifest itself as an acquired disease or as a component of the autoimmune polyendocrinopathy-candidiasis-ectodermal dystrophy syndrome (APECED).

Other secondary causes of HypoPT can be due to treatment with head and neck radiotherapy, malabsorption, alcoholism, or other states of malnutrition that result in magnesium depletion [6,7,9]. In fact, hypomagnesemia, decreasing PTH secretion, induces peripheral PTH resistance and mimics/increases hypocalcemia symptoms (i.e., confusion or memory loss, muscle spasms, numbness and tingling in the hands, feet, and face, depression, hallucinations, etc.).

There is another cause for HypoPT that is extremely rare. This is iron accumulation in the parathyroid glands in patients with thalassemia or hemochromatosis, copper accumulation in Wilson’s disease, patients with granulomatous disease, or metastatic infiltration of the parathyroid glands [6,7,9].

**Figure 1:**
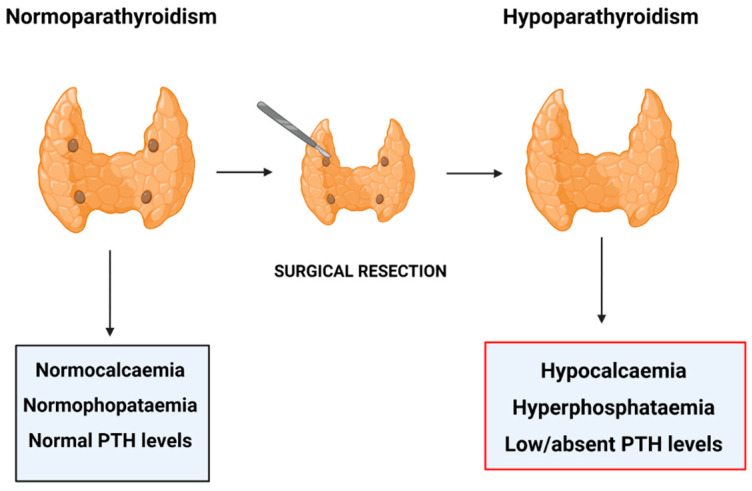
Post-surgical Hypoparathyroidism. Figure created in BioRender.com, accessed on 21 September 2021.

Post-surgical HypoPT is the most frequent form of acquired HypoPT (Figure 1). The most frequent form of HypoPT (~80%) is post-surgical hypoparathyroidism, due to the removal or damage of the parathyroid glands during neck surgery (secondary HypoPT) [7,9].

Unintentional damage by devascularization, thermal damage, or the removal of the parathyroid glands during neck surgery are the most frequent causes of this type of HypoPT. The incidence of post-surgical HypoPT is highly variable and depends on the degree of experience of the surgical center. Transient HypoPT is common after neck surgery. However, hypoparathyroidism that lasts more than six months after surgery is almost certainly considered chronic. Transient and permanent post-surgical HypoPT occurs between 5.4–9.6% and between 0.5–1.9% of cases, respectively [12], and clinical manifestations are related to the severity and chronicity of hypocalcemia.

As stated above, damage or complete removal of the parathyroid glands leads to insufficient PTH secretion and, consequently, to low calcium serum levels.

Once HypoPT is diagnosed, it’s treated by the administration of calcium and active vitamin D (e.i. calcitriol) or the administration of the parent vitamin D (cholecalciferol or ergocalciferol). In patients that also have hypercalciuria, thiazidediuretics are considered useful [6,12]. The primary goals for the management of HypoPT are to prevent symptoms of hypocalcemia; to maintain serum calcium concentration in the low range or even 0.5 mg/dL below the range (4.4 mmol^2^/L^2^); to avoid renal calcification (nephrocalcinosis/nephrolithiasis) and other extra skeletal calcifications; and, finally, to avoid the occurrence of hypercalciuria and hypercalcemia [6].

Currently, in selected cases of patients not responding to conventional therapy, daily injections of PTH peptides may be prescribed, in order to balance the low levels of calcium in the blood [6,14].

The clinical manifestations of hypofunctional disorders are often insidious and nonspecific, and lifelong hormone replacement therapy may sometimes be insufficient to correct metabolic disorders. Organ or cell transplantation is considered an alternative physiological approach to the treatment of endocrine system disorders of various metabolic conditions. Human and animal experiments have been conducted on endocrine organ transplantation for the treatment of endocrine diseases, such as (HypoPT).

In relation to this, in the last years several in vivo experiments have been realized to find new strategies to develop new methods of treatment for endocrine disease.

The principal aim of this review is to examine the current state of the art of cell and tissue therapy in HypoPT and to evaluate which types of therapy could be valid for future clinical use.

## 2. Transplantation Technologies and HypoPT

In endocrinology, transplantation can be performed using a whole endocrine gland (such as for the pancreas [15,16]), endocrine tissues (such as parathyroid tissue [17,18]), or endocrine cells (such as for pancreatic islet cell transplants [19,20]). The transplants can be autologous (the patient’s own tissues), syngenic (in genetically identical subjects such as monozygotic twins), or allografts (in subjects genetically dissimilar to the donor’s tissue). The allotransplantation of an organ or tissue from a donor to the body of a genetically compatible recipient has had limited success in the field of endocrine disorders [21,22,23,24], mainly due to rejection caused by the response of the Major Histocompatibility Complex (MHC). In the case of transplantation, in fact, there could be a direct recognition of the donor’s foreign MHC molecules through Antigen-Presenting Cells (APC) and recognition by the recipient’s T cells. Donor MHC molecules can be processed and presented to the recipient’s MHC complex and, consequently, recognized as foreign. These phenomena underlie the rejection of the transplant, the host’s disease towards the donor, and the reaction towards cancer cells [25,26,27].

Systemic immunosuppression for allograft recipients is a standard therapy that is performed for a few months after transplant, but it can also be taken throughout the patient’s life. Such therapy is not fully justified because immunosuppressants themselves often exert direct toxicity on endocrine cells [1]. Therefore, even if endocrine cell or tissue transplantation is an attractive alternative to whole organ transplantation, a complete immune tolerance has not yet been achieved [22,28,29,30,31,32] and more investigations and experiments are needed.

The methodology of the autotransplantation for parathyroid tissue is a standard technology used in postsurgical HypoPT. In this following section, we will review the various transplantation approaches used in HypoPT.

### 2.1. Autotransplantation

Lahey and colleagues performed the first parathyroid autotransplant following a thyroidectomy in 1926 [33]. Over the following 50 years, this procedure was shelved, until Wells and colleagues [34] repeated it clinically, physiologically, and histologically, by creating functional autografts.

There are various techniques for autograft [17,34,35,36]. Today, one of the most commonly used methods is that outlined by Wells and colleagues [34]. They propose, as soon as the excision is done, to incubate the parathyroid tissue in saline solution at 4° for 30 min, in order to allow the tissue to cool. The parathyroid tissue sample becomes solid enough to be cut into slices or cubes approximately 1mm thick. Then, 10 to 20 pieces are grafted into the chosen skeletal muscle. There are different possible anatomic areas in which to transplant the glandular tissue, such as the sternocleidomastoid, brachio-radialis, pectoralis major [37], and subcutaneous tissue of the forearm of the non-dominant arm [33,38]. Another method is the injection of a suspension of parathyroid tissue in buffered saline into the sternocleidomastoid muscle. Hicks and colleagues [39] in their study used 1–2 mL sterile physiological solution, minced the de-vascularized parathyroid gland, and suspended the obtained parathyroid fragments in a sterile solution of Dulbecco’s phosphate—buffered saline (DPBS) to obtain a tissue suspension. After that, the obtained suspension was injected sterilely via a sterile syringe into the right sternocleidomastoid muscle at the level of its midpoint.

In 2018, a new classification of the parathyroid glands was published to permit surgeons to evaluate, during thyroid surgery, which parathyroid could be easily preserved in situ and which must be autotransplanted [40]. Su and colleagues, in their study, proposed a classification strategy, recommending the autotransplantation only of parathyroid glands located inside the thyroid capsule or in the thyroid parenchyma, while they suggested preserving the others in situ.

They also reported an important aspect, namely that the incidence of transient post-operative HypoPT is higher in patients treated with the traditional method than in patients treated following the new classification of parathyroid glands, demonstrating that the correct preservation or autotransplantation of the parathyroid glands helps to minimize the incidence of permanent HypoPT [40].

Several studies have shown the possibility of permanent HypoPT and hypocalcemia following autotransplantation, as, i.e., in the study by Tartaglia and colleagues [41]. Tartaglia and colleagues [41] reported their experience of parathyroid transplantation during thyroidectomy to see whether grafting could influence the rate of postoperative hypocalcemia/HypoPT. They screened three groups of people: group A, 57 patients who underwent autotransplantation; group B, 87 patients who did not undergo autotransplantation, but experienced an accidental excision of a parathyroid gland; and group C, 100 patients who underwent autotransplantation without accidental removal of parathyroid glands. The incidence rate of HypoPT was 3.5%, 3.45% and 1% in groups A, B and C, respectively. Consequently, they could state that there were no significant differences in the parameters analyzed between groups A and B, suggesting that parathyroid autograft does not affect the rate of postoperative hypocalcemia and/or HypoPT. The same results were found in several other retrospective studies [42,43,44,45,46,47,48,49,50,51].

Conversely, in 2018, Su and colleagues [52] evaluated that autotransplantation of a parathyroid gland does not affect the incidence of permanent HypoPT, but it could increase the risk of transient HypoPT if the rest of the other glands are preserved in situ. On the basis of their observations, they suggested the preservation of at least two parathyroid glands during neck surgery in order to prevent transient HypoPT.

In the same year, Zhang and colleagues [53] published a study about the identification of the predictor factors of graft function after a parathyroid autotransplantation carried out during a thyroidectomy. They recorded PTH levels, patient age, sex, extent of surgery and postoperative serum calcium levels. They noticed that low serum calcium levels in the early postoperative period may stimulate a functional graft recovery, suggesting the choice of a moderate postsurgical calcium supplement strategy in patients selected for parathyroid autotransplantation [53].

In recent years, parathyroid autotransplantation has repeatedly been found to be a risk in terms of transient hypocalcemia [18,44,50,54,55,56,57,58,59]. Edafe and colleagues [57], in their meta-analysis evaluating predictors of post-thyroidectomy hypocalcemia, demonstrated that autograft patients were doubly at risk to develop transient hypocalcemia.

Parathyroid autotransplantation represents an interesting therapeutic approach for patients affected by secondary hyperparathyroidism (sHPT) [60,61,62,63,64], a disease often caused by end-stage renal failure and complicated by metabolic bone disease, severe atherosclerosis, and unwanted cardiovascular events [65]. Nowadays, parathyroidectomy is performed in 5–10% of all the diagnosed cases of sHPT [63] and in 10–30% of patients with more than 10 years of hemodialysis [64]. Both subtotal parathyroidectomy and total parathyroidectomy could be followed by autotransplantation in patients affected by sHPT [66].

More recently, Zhang and colleagues [61] and Du and colleagues [62] described a new surgical technique for parathyroid autotransplantation. Zhang and colleagues [61] described that six patients with sHPT were treated with endoscopic parathyroidectomy followed by autotransplantation. In this technique pieces of removed parathyroid gland were cut into cubes, inserted into a metal sleeve, and then squeezed into a syringe. After squeezing, the pieces were gently mixed with a sterile DPBS solution and injected into brachio-radialis without formation of an arteriovenous fistula for hemodialysis. The preparation time of this squeezing technique was less than 10 min for all patients. Du and colleagues described the use of the same technique in another group of 15 patients who were affected by sHPT. Both the research groups observed that after this type of autotransplantation, the preoperative symptoms were alleviated, serum parathyroid hormone and alkaline phosphatase levels, and hyperphosphatemia and hypercalcemia, were improved or normalized in all the patients of both groups. These positive effects have been confirmed to be linked to the fact that, as demonstrated by pathological examinations, parathyroid cells remain functionally active after autotransplantation.

### 2.2. Allotransplantation of Parathyroid Glands

An alternative treatment for patients affected by permanent HypoPT could be the allotransplantation of cultured parathyroid cells, combined with a short-term immunosuppressive therapy.

In 1975 Well and colleagues [34] performed and described the first parathyroid allotransplantation. An immunosuppressed parathyroid patient received a parathyroid allograft from a parent who previously had been his renal transplant donor. They observed that after a 30-month follow-up, the graft remained perfectly functional, and it secreted hormones and maintained normal serum calcium levels. Nevertheless, the manifestation of side effects related to the necessary lifelong immunosuppression therapy had a negative impact on the outcome of this therapy.

Over the following years, several other studies were carried out to try to find a way to solve the histocompatibility problem with the final aim to reduce/prevent the need for immunosuppression therapy. It was discovered that the biggest part of the parathyroid tissue presents the Human Leucocyte Antigen (HLA) class I only slightly expressed, so parathyroid cells may not be involved in transplant rejection. It has been possible to develop a long-running allograft that excludes the cells that express HLA class II antigens (i.e., lymphocytes, macrophages and granulocytes) from the insertion into the recipient [22,32,67,68].

From 1996 [24] until now, allotransplantation techniques have improved. As proof of this, in the last 10 years, two new parathyroid transplantation techniques have been described.

In 2016, Agha and colleagues [32] published a case report of a 32-year-old female who underwent surgery for multinodular goiter in 2006. During the surgery, a papillary thyroid carcinoma was detected, so the thyroid and all the parathyroids were removed. Despite supplement therapy, five years after surgery the patient exhibited a persistent symptomatic and refractory hypocalcemia. Therefore, to try to solve this symptomatic condition, the patient underwent a parathyroid transplant. In this case, thanks to the interest expressed by her brother to be evaluated as a potential donor, and after his identification as ABO compatible to the recipient, a living-donor allotransplantation of parathyroid glands was performed. This was the first type of parathyroid transplant based on the fragmentation of the parathyroid glands. In the procedure described by Agha and colleagues [32], the parathyroid tissue was reduced into small fragments to be implanted in the recipient’s left forearm brachio-radialis muscle. The mandatory immunosuppressive therapy regimen was the same as followed in the case of kidney transplantation. Immunosuppression was initiated during surgery and before the implantation of parathyroid fragments with the administration of prednisolone (500 mg) and a preoperative single dose of basiliximab (20 mg). The administration of these drugs, before and during the allotransplantation, were followed by the administration of tacrolimus six hours after surgery. They also reported that five days after transplantation the patient received a second dose of basiliximab (20 mg). The administration of prednisolone was gradually reduced to a maintenance dose of 2.5 mg per day after six months. Hormonal replacement therapy with (PTH) was stopped after transplantation. In this study, the donor showed normal calcium and PTH levels after surgery. At the same time, the recipient showed an increase of PTH serum levels (21 pg/mL) which remain (see Table 36) months after transplantation [32].

A similar technique, followed by a different immunosuppression therapy, was described by Yucesan and colleagues [22]. The patients received 250 mg methylprednisolone one hour before the planned transplantation and 60 mg of methylprednisolone on the postoperative day to minimize the host reaction. Oral prednisolone 5 mg per day was prescribed for one week only. In this case, they did not see any complications during the follow-up period, demonstrating that a fresh-tissue parathyroid transplantation associated with a short-term immunosuppression therapy could represent a promising technique, easy to perform, and with a low risk of side effects and minimal complications with compatibility for the HLA condition. Another type of parathyroid gland transplantation is the allotransplantation of cryopreserved parathyroid tissue [67,68,69,70,71,72,73].

In relation to the development of new parathyroid allotransplantation methods, Aysan and colleagues [67,68] described a new parathyroid allotransplant method which has been described in several other studies [74,75,76,77,78,79,80]. This new method is based on the separation of the collected donor parathyroid tissue from the surrounding blood vessels, gland capsule, connective, and fatty tissue through a sterile filtration, followed by a mechanical fragmentation of the isolated tissue in 1X DPBS solution supplemented with 5% inactivated fetal bovine serum. All these procedures must be performed under sterile conditions. The obtained suspension was filtered into a 15mL tube using a sterile cell strainer and treated with 600 µL of deoxyribonuclease I. It was then centrifuged at room temperature to obtain a pellet, which was suspended in cell culture medium. Upon evaluation of the cell viability, cells were cryopreserved until transplantation. For the grafting, they used 50 million cells, and the transplant was performed via intramuscular injection in the left arm deltoid muscle. As immunosuppressive therapy, prednisolone was administered from five days to 1 month (depending on the study) and then stopped. In both of their studies, Aysan and colleagues [67,68] reported no side effects and a success rate of 70% at a mean follow-up of 12 months.

There are studies, like one published by Flechner and colleagues [69], showing that the kind of grafting described by Aysan and colleagues does not always work after the first transplant, and a second one is sometimes necessary. In their study, they reported that, in 2010, a patient was transplanted using eight months cryopreserved parathyroid tissue by an injection in the left brachioradialis muscle. The transplant was followed by the administration of an immunosuppressive therapy with tracolimus, mycophenolate mofetil, and steroids. Two weeks after the transplant, they reported that the recipient felt an increase of pain, and after two months the PTH serum level was <10 pg/mL. Therefore, the patient underwent a second allotransplant from the same donor, and eight months later he became clinically asymptomatic.

Regarding the studies described previously, it is stated that cryopreserving parathyroid tissue can contribute not only to the loss of surface HLA expression, but also to the reduction of immunogenicity. Both these aspects are confirmed by the observation that in all the parathyroid transplants through the cryopreserved tissue, immunosuppressive therapy has only been necessary for a short period. This means that the side effects of an anti-rejection life-therapy could drastically decrease.

Nevertheless, this other type of parathyroid transplant presents some problems related to cryopreservation and the number of cells necessary to have a successful outcome.

Several studies have reported that about 70% of the cryopreserved tissue is viable in the first 24 months after preparation and that cells are viable after thawing. They have also reported that cell viability is indirectly proportional to the period of cryopreservation [34,56,57,58,59,60,61,62,63,64,65,66]. In relation to these observations, although a large amount of excess parathyroid tissue is usually frozen for future grafting, it is mandatory to know the quantity of parathyroid tissue sample required for a good outcome without risking complications after parathyroid allotransplantation. Regarding the cryopreservation method, several researchers described a standard method of tissue manipulation and gradual preparation for freezing and storing in liquid nitrogen, which have not changed significantly over time [73,74,75,81,82].

### 2.3. Microencapsulation Techniques and Parathyroid Glands

**Figure 2:**
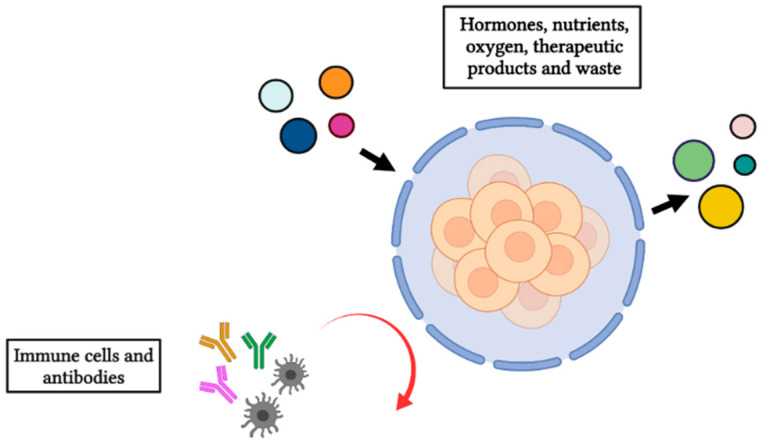
Illustration of the semipermeable membrane allowing the bidirectional diffusion of nutrients, oxygen, therapeutic products, and waste, while at the same time avoiding the entrance of immune cells and antibodies. Figure created in BioRender.com, accessed on 21 September 2021.

The technique that has the potential to revolutionize the treatment of endocrine disorders is microencapsulation [83], an innovative approach which consists in encapsulating cells with a coating created with a chosen biomaterial that has the characteristic of being selectively permeable [83]. The chosen biomaterial must present a selective permeability which is not only able to mimic the physiological microenvironment but is also able to limit the attack of the host immune system by excluding large molecular weight components, such as antibodies and immune cells (Figure 2).

At the same time, the spatial characteristics of the biomaterial promote a high diffusion rate, allowing for hormone secretion, cellular oxygenation, and nutrient penetration [84]. This also significantly affects the long-term viability of encapsulated cells. In recent years, several types of biocompatible hydrogels have been tested for microencapsulation, and of these microencapsulation systems, alginate (ALG) has shown the best results [85].

ALG, a polysaccharide naturally derived from algae, is composed of the irregular blocks of β-D-mannuronic (M) and α-L-guluronic (G) acids with various compositions and sequential arrangements [86]. Strength, brittleness, rigidity, and softness of the ALG gel are closely related to the block number and length of the G block regions in the mix [87].

One of the most important properties of sodium alginate is its ability to form hydrogels. The manufacture and design of hydrogels must be perfectly executed to obtain ALG-based hydrogels that can be injectable at room temperature or even lower; in addition, they must be biodegradable, biocompatible, and suitable as a support for cell induction. Since this natural polymer is known to be extensively contaminated [83], several purification methods have also been developed to improve the biocompatibility of the ALG hydrogel. Purification of the ALG not only improves the biocompatibility of the microcapsule, but also avoids the development of an overgrowth of fibrotic cells around the microcapsule, which is common when using impure natural ALG [88,89,90].

This technique has the possibility of changing the future of therapies for endocrine disorders, making it possible to perform allografts through microencapsulation, as well as autologous transplants.

The first microencapsulation experiments to investigate if this technique could be used as a possible treatment for endocrine disorders was performed by Lim and colleagues in 1980. In their work they describe the preparation of sodium alginate microcapsules in which they incapsulated Langerhans islets as a possible future therapeutic strategy to cure for type 1 diabetes mellitus (T1D) [91]. After this study, the microencapsulation technique applied to T1D has been improved over the past 40 years with the final aim to make the microencapsulation of pancreatic islets the gold standard transplantation strategy to treat T1D [20,28,92,93].

On the bases of previous and recent studies on the use of microencapsulation as a therapeutic strategy for pancreatic related disorders, researchers started to hypothesize about how to use this technique to treat other endocrine disorders, such as HypoPT, hypothyroidism, hyposurrenalism and hypopituitarism.

The microencapsulation technique applied to transplantation practice could represent, in the case of parathyroid allotransplantation, an interesting and successful technique, given that it avoids immunity system activation and consequent side effects.

Several types of artificial materials have been tested to obtain a good outcome for the microencapsulation technique, such as sodium polystyrene, chitosan, PLGA-alginate, poly-L-ornithine, polyethylene glycol, collagen, cellulose, polylisine, photosensitive poly (L-lysine), mitogenic or amitogenic alginate, polyvinylidene difluoride, and calcium chloride [76,77,78,79,80,94,95,96,97,98,99]. Among all these materials, ultrapure alginate was proven to be the best-qualified material for human studies, not only for the biocompatibility of the material, but also because it prevents rejection correlated to the activation of the host immune system. This technique has been improved through numerous studies since the late 1980s [78]. In that period, the question was to understand how cell microencapsulation could be done to preserve cell viability and endocrine functionality [77,78,79,80,94,95,96].

To respond to this important point, Hasse and colleagues [95] showed that isotransplanted parathyroid tissue remains vital and functioning in vivo over long periods of time using a novel amitogenic alginate, and not the mitogenic one.

Picariello and colleagues [76] demonstrated that encapsulated human parathyroid cells (5 × 10^6^ cell per microcapsule) maintained their integrity for over three months in culture, and that the percentage of viability was 90%, 70%, and 60% at 48 h, 15 days, and three months, respectively. They also reported that the secretion of PTH was stable for over three months and was higher than the PTH secretion levels measured in cell culture medium under normal monolayer growth conditions.

Chen H.L. and colleagues [79] tried to microencapsulate human parathyroid cells into a three-layer structure that consisted of alginate/photosensitive poly(L-lysine)/short chain alginate-co-MPEG. In the in vitro studies, they observed that the microencapsulated parathyroid cells maintained differentiative proprieties in culture, and the capsular membrane was perfectly permeable to the PTH. In vivo studies on the transplant of these encapsulated parathyroid cells into parathyroidectomized SD-rats observed that the serum calcium levels were restored after transplantation. In addition to this, the successive histological evaluation of the excised transplanted microcapsules showed that the microcapsules remained perfectly structurally intact and were free of cell adherence.

In 2007 Moskalenko and colleagues [80] assessed the stability of different types of microcapsules, one with tissue particles and the other with single cell/cell clusters, using an osmotic pressure test. Their analyses revealed that tissue particles generated deformed and imperfect capsules which had poor stability compared with single cell/cell cluster capsules.

In relation to the use of microencapsulation, Cabané and colleagues [96] published a case report about an allotransplantation of parathyroid tissue in a patient with severe HypoPT who previously received continuous endovenous supplementation of calcium as therapy. At the beginning, they implanted 23 microspheres in the non-dominant forearm. After 24 h after grafting, because serum calcium and PTH levels had increased (9.6 mp/dL and 5 pg/mL, respectively), they discontinued intravenous calcium therapy. From day 3 until day 30 after surgery, PTH levels dropped to a value of 1 pg/mL; thereafter, levels increased to 4.8 pg/mL To obtain a normal serum level of PTH, they performed a second allotransplant with 40 more microcapsules implanted into the right leg. Unfortunately, the second attempt failed, and after 21 months it was necessary to resume calcium supplementation therapy.

The allotransplant performed and described by Yucesan and colleagues [98] had a different outcome. They transplanted 689 microcapsules in 2% ultrapure calcium chloride ultrapure alginate in the omental tissue of a patient affected by severe permanent HypoPT.

Ten days after the transplantation it was decided to stop the therapy in order to evaluate the real effects of the use of microcapsules as described in this paper. The controls performed at the various follows-ups showed that not only did serum calcium levels remain stable over the time (7.2–9.1 mg/dL), even at one year after the first follow-up, but that PTH serum levels were also positively increased (from 1.3 to 8.4 pg/mL).

At this point, based on the studies analysed here, it is possible to state that cell microencapsulation is not only the best technique to avoid immune response, but it is also extremely suitable as a parathyroid transplantation technique (~74% of success rate) [98].

Nevertheless, this technique is not yet fully defined for use as a clinically recognized possible treatment for HypoPT. This is related to the fact that we still do not know with certainty which is the correct and sufficient number of microcapsules to be used in parathyroid transplantation in order to successfully treat HypoPT, as well as the number of cells to be inserted into the microcapsules to ensure the regeneration of damaged parathyroid glands. Finally, from the studies analysed here, it is necessary to evaluate which is the best anatomical site for the insertion of microcapsules, especially to ensure the survival of the cells presented in them, ensuring the success of parathyroid tissue transplantation. Therefore, future studies are necessary in order to use parathyroid transplantation trough microencapsulation as a recognized treatment for HypoPT.

### 2.4. Stem Cell Therapy as Treatment for HypoPT

Stem cell therapy represents a therapeutic revolution to replace pharmacological treatments, and in the last decades has been the principle subject of numerous studies for several diseases (i.e., osteonecrosis [100], diabetes mellitus [101], and nervous diseases [102,103].

The use of stem cells could represent an important step forward in the regeneration of damaged parathyroid tissue.

Ignatoski and colleagues and Bingham and colleagues [104,105] investigated the possibility to generate in vitro a cellular model which is able to produce PTH starting from mouse embryonic stem cells and from human Embryonic Stem Cells (hESCs), respectively. They used Activin A and soluble Sonic Hedgehog factors to differentiate these embryonic stem cells into parathyroid-like cells.

Particularly, Ignatoski and colleagues [104] differentiated hESC cells into parathyroid-like cells by culturing them with a combination of increasing FBS and timed Activin A exposure. They reported that these cells express both differentiation parathyroid gene markers, such as Glial Cells Missing Transcriptor Factor 2 (GCM2), and differentiation genes, such as CaSR and PTH genes. They also reported the ability of these differentiated cells to produce and secrete PTH.

These collective results showed that differentiation of hESCs into parathyroid-like cells could be a potential technique to differentiate cells from patients into PTH-secreting cells, in order to use them for potential regenerative therapy. Other studies are being conducted following this line of research.

In 2009, Shih and colleagues [106] isolated and characterized parathyroid derived stem cells (PDSCs) directly from human parathyroid glands. They plated their specimens, after enzymatic digestion, with an average of 30 cells on each 96-well plate after limiting dilution. They obtained 55 proliferating clones from five donors; six of these differentiated into all three mesodermal cell types. Their results revealed that PDSCs were phenotypically similar to mesenchymal stem cells (MSCs). Moreover, these cells had a high telomerase activity, and could do chondrogenic, osteogenic and adipogenic differentiation.

Following these previous studies, Park and colleagues [107,108] also performed important studies on the implantation of scaffold-free parathyroid tissue spheroids using (for the first time) differentiated human tonsil derived mesenchymal stem cells (dTMSC). In their work they described the differentiation into parathyroid cells of dTMSCs and their use in restoring in vivo parathyroids functions. Data reported in their studies show that after 3threemonths the HypoPT was treated in mice, leading one to surmise as to whether the use and the manipulation of mesenchymal stem cells could be a feasible treatment for HypoPT and damaged parathyroid tissue.

## 3. Conclusions

In this review, we reported the history, several passages, and new studies that have led researchers to consider the use of biohybrid artificial implants as a potential therapy for HypoPT instead of autotransplantation, which leads to good outcomes, in most cases, even if it is not always possible to perform. Therefore, as observed in the literature analysed here, to avoid the side effects related to autotransplantation the researchers began conducting studies on the possibility of using the microencapsulation technique to solve HypoPT. Nevertheless, as noted in this study, this technique has not been defined, and more investigation is needed regarding biocompatibility, long-term stability, and interactions of the transplanted microcapsules with the surrounding tissue environment. In addition, the relative number and size of the microcapsules to be transplanted for an optimal outcome still needs to be determined.

Always searching for new experimental approaches for the treatment of HypoPT, especially given the recent rise of the topic regenerative medicine based on the use of stem cells, we notice that the research is moving in this direction for HypoPT as well. In conclusion, all of the recent studies reveal that the possible future and promising therapy for HypoPT could be a combination of stem cell therapy and the use of biohybrid artificial implants. This is the new frontier in the therapy of HypoPT which could pave the way for new modes of treatment of other several endocrine disorders and of all the diseases characterized by damage to an organ or to tissue.

## Data Availability

Not applicable.
